# Citicoline in acute ischemic stroke: A randomized controlled trial

**DOI:** 10.1371/journal.pone.0269224

**Published:** 2022-05-31

**Authors:** Ayush Agarwal, Venugopalan Y. Vishnu, Jyoti Sharma, Rohit Bhatia, Ajay Garg, Sadanand Dwivedi, Ashish Upadhyay, Vinay Goyal, Mamta Bhushan Singh, Anu Gupta, Roopa Rajan, M. V. Padma Srivastava

**Affiliations:** 1 Department of Neurology, All India Institute of Medical Sciences, New Delhi, India; 2 Department of Neuroradiology, All India Institute of Medical Sciences, New Delhi, India; 3 Department of Biostatistics, All India Institute of Medical Sciences, New Delhi, India; Universita degli Studi Magna Graecia di Catanzaro, ITALY

## Abstract

**Introduction:**

Two pharmacological possibilities exist for an acute ischemic stroke (AIS): recanalization of the occluded artery and neuroprotection from ischaemic injury, the latter’s efficacy being debatable. We sought to determine whether administration of Citicoline immediately after recanalization therapy for AIS would improve clinical and radiological outcome at three months compared to standard treatment alone.

**Patients and methods:**

CAISR was a single centre, randomized, placebo-controlled, parallel-group trial with blinded endpoint assessment. It was approved by the All India Institute of Medical Sciences Institutional ethics committee and registered at the Clinical Trial Registry of India (CTRI/2018/011900). We recruited participants with AIS undergoing recanalization therapy and randomly assigned them to receive either Citicoline or placebo in 1:1 ratio. Citicoline arm patients received Citicoline 1gm BD intravenously for three days, followed by oral citicoline 1gm BD for 39 days. Placebo arm patients received 100ml intravenous normal saline for three days, followed by multivitamin tablet BD for 39 days. All patients received standard of care.

**Outcome:**

Blinded assessors did the follow-up assessment at six weeks (MRI Brain-stroke volume) and three months (NIHSS 0–2, mRS 0–2 and Barthel index> = 95).

**Results:**

The infarct volume decreased from week 1 to week 6 by 2.6 cm^3^ on placebo versus 4.2 cm^3^ on Citicoline (p-0.483). The OR for achieving NIHSS 0–2, mRS 0–2 and Barthel index> = 95 with Citicoline was found to be 0.96(95%CI 0.39–2.40), 0.92(95%CI 0.40–2.05) and 0.87(95%CI 0.22–2.98) respectively.

**Conclusion:**

CAISR was the first to evaluate the role of Citicoline, when used immediately after recanalization therapy, when the penumbral tissue is the most susceptible either to be protected from injury or become ischemic. We did not find any significant difference between the Citicoline or placebo arms with respect to either our primary or secondary outcomes.

## Introduction

Stroke is one of the leading causes of mortality and morbidity worldwide [1, 2]. Stroke being a medical emergency, outcomes often depend upon how quickly the patient is evaluated and treated since "time is brain". Two potential pharmacological possibilities exist for an acute ischemic stroke: rapid and complete/near-complete recanalization of the occluded artery and protection of the brain parenchyma from ischaemic injury [3]. The former exists presently as thrombolysis with either Alteplase or Tenecteplase within 4.5 hours of stroke onset [4] while the latter’s efficacy is still debated.

Recent research in animal models has suggested that neuroprotectant drugs can enhance repair and endogenous brain plasticity, reducing acute brain damage and improving functional recovery after an acute ischemic stroke [5, 6]. Citicoline is one such neuroprotectant drug. It is an exogenous form of cytidine 5ʹ-diphosphate choline, an essential intermediate for the generation of phosphatidylcholine and sphingomyelin, required for membrane biosynthesis. During ischemia, this membrane gets degraded into free radicals and fatty acids [7]. Citicoline gets broken down by CTP: phosphocholine cytidylyltransferase (CCT) into choline and cytidine triphosphate (CTP), which then crosses the blood-brain barrier and helps in the generation of phosphatidylcholine, thereby preventing membrane breakdown. Experimental models have revealed its action at other levels of the ischemic cascade as well [8]. These include decreased glutamate levels and caspase activation products, thereby inhibiting ischemia induced neuro-inflammation and its induced apoptosis [8].

Citicoline has been extensively studied in over 1100 patients enrolled in clinical trials of various neurological disorders, including acute stroke and was found to have a safety profile similar to placebo [8]. However, it showed no efficacy in the primary end-points [9–11] and only revealed positive outcomes in some studies’ post-hoc analysis.

A meta-analysis on Citicoline in 2002 for acute/subacute stroke care [12] suggested a beneficial treatment effect, with a 10–12% absolute reduction in long-term death and disability. It was found to have an OR of 1.33 (95% CI = 1.10–1.62) compared to placebo, for complete functional and neurological recovery, when used for six weeks in patients with moderate to severe ischemic stroke [7]. Another updated meta-analysis confirmed these results, as it found a reduction in death or dependency with its use (OR 0.65, 95% CI = 0.54–0.77; *P* = .00001; NNT = 9.5) [13]. However, its efficacy over and above the best standard of care, could not be proven in the recently concluded randomized controlled ICTUS trial [3].

However, Citicoline has never been studied in the setting of acute ischemic stroke patients undergoing endovascular thrombectomy. Moreover, even in patients receiving intravenous thrombolysis, it was never studied in the immediate peri-intervention period. We sought to determine whether the administration of Citicoline immediately after recanalization therapy for acute stroke would improve clinical outcome and radiological outcome at three months compared to standard treatment alone.

## Methods

### Study design and participants

The Citicoline In Acute Ischemic Stroke (CAISR) trial was a single centre, randomized, placebo-controlled, parallel-group trial with blinded endpoint assessment. We recruited participants with acute ischemic stroke undergoing recanalization therapy from Emergency services and Neurology Inpatient services at a tertiary hospital in India who presented in the period between May 2017 to September 2020. The All India Institute of Medical Sciences Institute Ethics Committee approved the study protocol. The trial was registered at the Clinical Trial Registry of India (CTRI/2018/011900). Written Informed consent was taken from all participants or their authorized representatives. The trial was conducted following the Declaration of Helsinki and the principles of Good Clinical Practice.

### Inclusion criteria

- Age > = 18 years
- Suspected acute ischemic stroke based on clinical and radiographic evidence as determined and documented by the stroke team
- Participants must meet criteria for intravenous thrombolysis or endovascular thrombectomy as determined and documented by Stroke Neurologist and Interventional Neuroradiologist, and receive either or both of these therapies
- Signed informed consent

### Exclusion criteria

- Intracranial haemorrhage
- Known allergic reaction to citicoline or contrast allergy
- CT or MRI evidence of brain tumour
- Previous disorders that may confound the interpretation of the neurological scales
- Pre-existing dementia, when dementia implies a disability, measured as a score of 3 or higher in the previous mRS
- Patients under current treatment with Citicoline

### Randomization and allocation

The participants were randomly assigned with concealed allocation in 1:1 ratio to receive either Citicoline or placebo immediately after the recanalization therapy. We randomly assigned patients into citicoline and placebo arms, using stratified variable block computer generation random table. Stratification was done for patients undergoing thrombolysis alone and thrombolysis plus endovascular thrombectomy (random number of 100 patients for thrombolysis and 24 patients for thrombolysis plus thrombectomy group was generated based on our sample size). Outcome assessors were masked to treatment allocation.

### Intervention

Citicoline arm patients received intravenous Citicoline 1gm (in 100ml normal saline) immediately after the recanalization therapy followed by 1gm BD intravenously for three days, followed by oral citicoline tablet 1gm BD for 39 days (total six weeks). Placebo arm patients received 100ml intravenous normal saline given intravenously for three days, followed by oral multivitamin tablet BD for 39 days. All patients received standard of care.

### Outcome assessment

Blinded assessors did the follow-up assessment at six weeks (MRI Brain-stroke volume) and three months (NIHSS, mRS and Barthel index). MRI brain included T1, T2, 3D-FLAIR, DWI and ADC sequences. Stroke volume was calculated on FLAIR images using 3D reconstruction on Reformat using MRI-AW VolumeShare5 software on Hewlett-Packard machine. All imaging analyses were done by a radiologist blinded to treatment allocation. The primary outcome was change in stroke volume from baseline to 6 weeks after stroke onset. The secondary outcome measures were mRS 0–2 at 90 days, NIHSS 0–2 at 90 days, Barthel index > = 95 at 90 days, and mortality at 90 days. The safety outcomes were death and adverse events.

### Workflow

**Fig 1:**
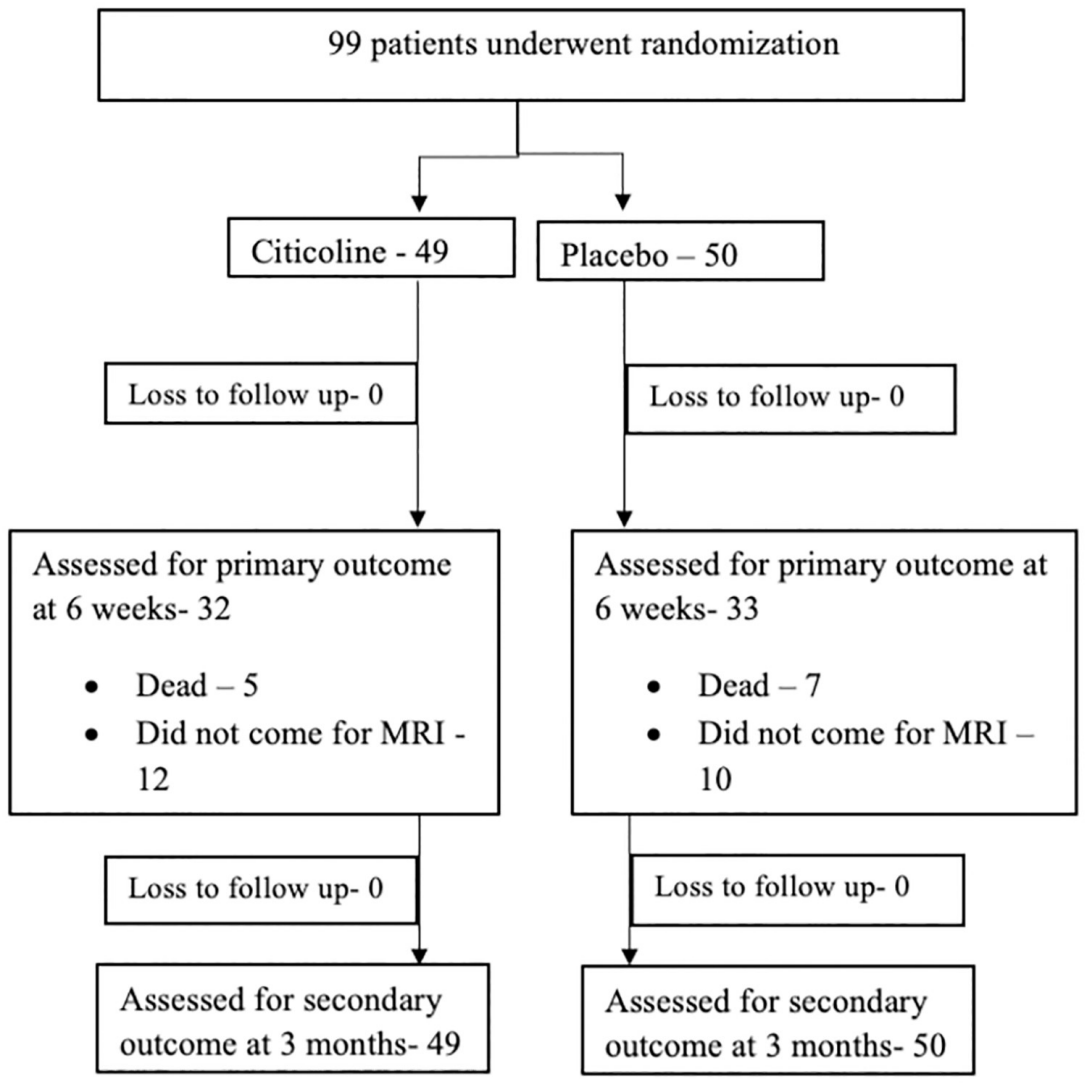
Consort flow diagram.

All acute ischemic stroke patients were screened, and those satisfying inclusion criteria were recruited. All patients underwent an NCCT head, and CT angiography and patients received one or more of the recanalization therapies (Alteplase/ Tenecteplase/ Mechanical Thrombectomy) before recruitment and randomization. The Tenecteplase used was the Tenecteplase analogue approved by the Drug Controller General of India. All patients received Citicoline or placebo as per protocol immediately after the recanalization therapy. Blinded assessment of mRS and NIHSS scores was done at three months. The patient underwent two MRI scans of the brain with infarct volume calculation: at admission and after six weeks. Stroke subtyping was done according to the TOAST classification for all patients (Fig 1).

### Sample size and statistical analysis

Based on the means and variances of the study by Warach et al., we assumed that lesion volume would decrease in 40% of the placebo arm and 65% of the citicoline-treated patients. We estimated that 116 (58 per group) completed patients (baseline and week six MRI assessments completed) were required to detect this difference with 80% power and a significance level of 0.05. No interim analysis was planned during the study.

The analysis was conducted in Stata version 13 (StataCorp LP, College station, TX, USA). Statistical analysis was performed using a chi^2^ test for categorical variables; t-test and Wilcoxon rank-sum test for continuous variables. Continuous variable not following standard distribution were analysed with Mann Whitney test. Statistical analyses for the primary endpoint were done based on the logistic regression model. All patients who died or were lost to follow up were given worst scores for both primary outcome measures at follow up (i.e. NIHSS-42 and mRS-6). Values in absolute numbers as well as in percentages were compared between two groups. Results were considered to be statistically significant if the P-value was <0.05.

## Results

Ninety-nine consecutive acute ischemic stroke patients were enrolled in CAISR. The baseline characteristics are mentioned in Table 1. The median age (in years) was 61 and 54.5 respectively, in the Citicoline and placebo groups. The majority were males (61.2% Vs 60%) in the two groups. Both groups had predominantly left-sided infarcts (Citicoline group-51% and placebo group-64%) and MCA territory involvement (81.6% Vs 84%). Both groups had similar blood pressure and ASPECTS score at presentation. The Citicoline arm patients presented earlier to the emergency than those in the placebo arm (135 minutes Vs 162.5 minutes). More patients underwent endovascular treatment in the placebo arm (16%) than the Citicoline arm (14.3%). Small vessel disease causing stroke was the commonest identified stroke subtype (47% Vs 52%), and hypertension was the most common risk factor identified in both groups. Patients in the placebo arm presented with milder strokes (median NIHSS-7.5) when compared to the citicoline arm (median NIHSS-10) but the duration of hospital stay was the same in both. None of these differences was, however, found to be statistically significant.

**Table 1:** Baseline characteristics.

Baseline Characteristics	Citicoline (n = 49)	Placebo (n = 50)	p-value
**Age (median) in years +/- SD**	61 +/- 14.5	54.5 +/- 14.6	0.333
**Males (%)**	61.2 (30/49)	60 (30/50)	0.901
**Infarct (%)**			0.419
• Right	49 (24/49)	36 (18/50)	
• Left	51 (25/49)	64 (32/50)	
**Vascular territory (%)**			0.694
• MCA	81.6 (40/49)	84 (42/50)	
• PCA	12.2 (6/49)	12 (6/50)	
• ACA	0	2 (1/50)	
• ICA	2.1 (1/49)	2 (1/50)	
• Watershed	4.1 (2/49)	0	
**Median ASPECTS**	8	8	0.883
**Median BP (mm Hg)**			
• SBP	150	148	0.309
• DBP	90	90	0.535
**Endovascular treatment (%)**	14.3 (7/49)	16 (8/50)	0.812
**Risk factors prevalence (%)**			
• Hypertension	55.1 (27/49)	62 (31/50)	0.486
• Diabetes mellitus	26.5 (13/49)	30 (15/50)	0.702
• Smoking	32.6 (16/49)	44 (22/50)	0.246
• Alcohol intake	22.4 (11/49)	22 (11/50)	0.957
• Dyslipidemia	8.2 (4/49)	10 (5/50)	0.751
• Heart disease	26.5 (13/49)	26 (13/50)	0.670
• Previous stroke	28.6 (14/49)	26 (13/50)	0.323
**Median GCS at presentation**	15	15	0.527
**Time frames (Median) in minutes**			
• Onset to hospital	135	162.5	0.139
• Onset to thrombolysis/EVT	165	200	0.244
• Onset of drug treatment	240	252.5	0.088
**Stroke subtypes- TOAST (%)**			0.546
• Large vessel	18.4 (9/49)	6 (3/50)	
• Cardioembolic	24.5 (12/49)	22 (11/50)	
• Small vessel	47.0 (23/49)	52 (26/50)	
• Other classified	0	0	
• Unclassified	10.1 (5/49)	20 (10/50)	
**Median hospital stay (days)**	6	6	0.931
**Baseline NIHSS (%)**			0.604
• <8	24.5 (12/49)	50 (25/50)	
• 8–14	51.0 (25/49)	26 (13/50)	
• 15–22	20.4 (10/49)	24 (11/50)	
• 23–42	4.1 (2/49)	2 (1/50)	
**Tenecteplase usage**	28.6 (14/49)	26 (13/50)	0.768

The median NIHSS at three months follow up was the same (four) in both arms. The median mRS at presentation and follow up were the same in both groups, being four and two respectively. The median stroke volume at presentation was slightly higher in the citicoline arm than the placebo arm (7.32 cm^3^ v/s 5.64 cm^3^ respectively). Median stroke volume measured at six weeks revealed 3.1 cm^3^ and 3.01 cm^3^ in the Citicoline and placebo arms, respectively (p = 0.79). However, we were only able to calculate the radiological outcome in 65 of the 99 patients as the remainder were unable to report for their follow-up MRI at six weeks due to the lock-down imposed because of the COVID-19 pandemic. We could not achieve our sample size as we had to stop our trial prematurely for the same reason.

**Fig 2:**
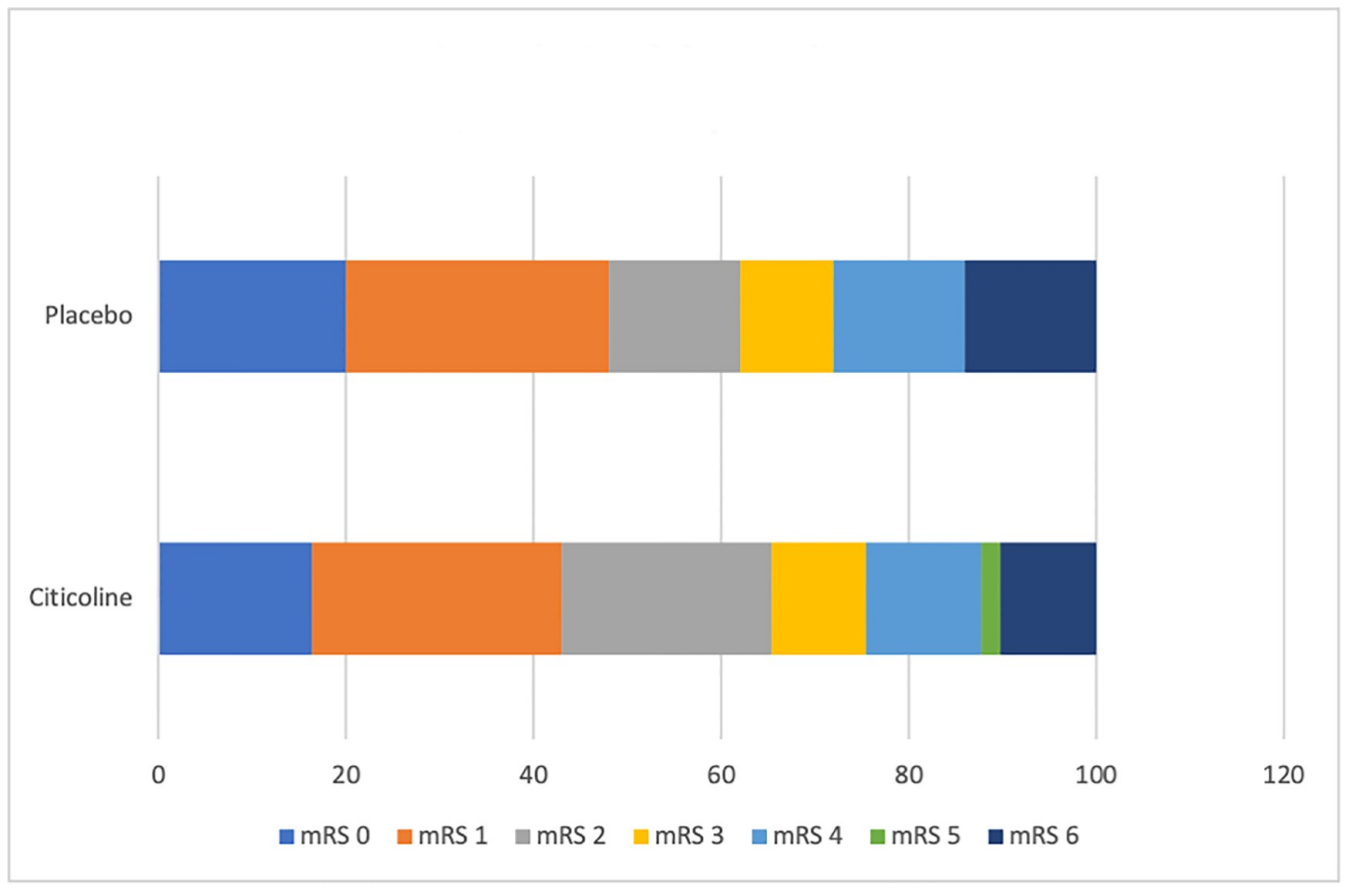
Shift analysis of the modified rankin scale (p value-0.700).

Favourable outcome after an acute stroke was defined as an NIHSS or mRS score between 0–2 at 3 months follow up or a Barthel index score of > = 95. Twenty patients in both arms fulfilled this criterion for NIHSS while thirty-two in the Citicoline arm and thirty-one in the placebo arm fulfilled it for mRS scores. Twelve patients died in CAISR (five in the Citicoline arm and seven in the placebo arm). Both primary and secondary outcome measures are mentioned in Table 2. No statistically significant difference was found in this outcome between the Citicoline and placebo groups. Shift analysis done for the mRS scores between the Citicoline and placebo groups was also not significant (Fig 2).

**Table 2:** Outcome measures (primary and secondary).

Outcome measures (Median)	Citicoline (n = 49)	Placebo (n = 50)	P-value	OR with 95% CI
**PRIMARY OUTCOME**				
**Change in stroke volume from baseline to 6 weeks (cm** ^**3**^ **)**	4.22	2.63	0.483	
**SECONDARY OUTCOME**				
**mRS 0–2 at 90 days**	32 (65.3%)	31 (62%)	0.732	0.92 (0.40–2.05)
**NIHSS 0–2 at 90 days**	20 (40.8%)	20 (40%)	0.934	0.96 (0.39–2.40)
**Barthel index > = 95 at 90 days**	8 (16.3%)	10 (20%)	0.564	0.87 (0.22–2.98)
**Mortality rate at 90 days**	10.2 (5/49)	14 (7/50)	0.468	

Adverse events were found to be equally prevalent in both groups with no statistically significant difference between the same. All adverse events are mentioned in Table 3.

**Table 3:** Adverse events.

Adverse events	Citicoline arm (n = 49)	Placebo arm (n = 50)	P-value
LRTI	6.1 (3/49)	10 (5/50)	0.573
Hemorrhagic transformation of infarct/ sICH	6.1 (3/49)	6 (3/50)	0.879
UTI	4.1 (2/49)	6 (3/50)	0.752
Hypotension	0	0	

## Discussion

"Time is brain" as nearly 2 million neurons die every minute after an ischemic stroke. Apart from reperfusion, preserving ischemic penumbra from further damage, using neuroprotectants is a major research target. Citicoline is one such neuroprotectant [14], which is widely used in stroke management but without proven efficacy.

Clark WM et al. conducted a phase II trial [9] with Citicoline in acute stroke patients and showed improved outcomes with doses of 500mg or 2000mg/day, but later in a similar phase III trial [10] found no difference in stroke outcomes. However, the post-hoc analysis revealed that a higher percentage of patients in the placebo arm had milder strokes and suggested improved outcomes with Citicoline in patients with severe strokes. Citicoline also showed a trend towards smaller infarct volumes [15]. Another phase III trial [11] enrolling only moderate-to-large strokes conducted with the novel endpoint of NIHSS improvement>7 points was also negative, which would have been positive if the usual outcomes of NIHSS and mRS score between 0–1 at three months been used. Davalos et al. [3] conducted a multi-centre, placebo-controlled randomized controlled trial that enrolled 2298 patients (1148 in Citicoline and 1150 in placebo arms) with acute anterior circulation ischemic strokes 24 hours of symptom onset. Randomization was done in a 1:1 ratio into Citicoline (1gm BD) and placebo arms, given for six weeks. No significant difference was found in the outcomes of NIHSS, mRS or Barthel index. The non-thrombolysed subgroup showed a positive tendency with Citicoline. Seccades et al. [16] conducted a systematic review and meta-analysis on Citicoline and found that its administration was associated with a significantly higher rate of functional independence, both by random and fixed effects model (OR-1.56 with CI 1.12–2.16 and OR-1.20 with CI 1.06–1.36 respectively), supporting its use in acute ischemic stroke, mainly when used within the first 24 hours. A systematic review conducted by Agarwal et al. [17] analyzed neurological, functional, cognitive and domestic adaptation evaluation in patients with stroke and traumatic brain injury treated with Citicoline. They found a significant beneficial effect on the functional outcome (OR-1.18, CI: 1.04–1.34). However, all other parameters showed an effect similar to placebo.

Despite all these studies on Citicoline, it was never studied in acute ischemic stroke patients undergoing endovascular thrombectomy. Moreover, even in patients receiving intravenous thrombolysis, it was never studied in the immediate peri-intervention period, when it is most likely to be effective. We had aimed to find out whether administration of Citicoline immediately after recanalization therapy for acute stroke will improve both radiological (decrease in infarct volumes at six weeks) and clinical outcomes (mRS and NIHSS of 0–2 and Barthel index > = 95 at three months) compared to standard treatment alone. However, it was found not to meet any of these primary or secondary endpoints.

When compared to the largest randomized controlled trial done on Citicoline, the ICTUS trial [3]

- The median age of CAISR was significantly younger at 60 +/-14.6 years, compared to 72.9+/-12.0 years
- CAISR enrolled more strokes of lesser severity as evidenced by approximately 75% patients having an NIHSS score of < = 14 at presentation compared to 47% in the ICTUS trial
- Thrombolytic agent was used in all patients in CAISR whereas it was used in 46% in ICTUS trial
- Small vessel disease was the most common cause of stroke comprising approximately 50% cases, whereas it constituted only 5% in the ICTUS trial. The latter had the cardio-embolic disease as the most common etiological cause of stroke, amongst the classified cases. The cardioembolic disease was second with an approximate percentage of 24% in CAISR
- High blood pressure was the most common identified risk factor comprising 60% prevalence in CAISR. The same was found in the ICTUS, but with a higher prevalence of 73%
- The prevalence of smoking was higher in CAISR with 35–40% active smokers compared to 16%
- Similar to it, all our baseline characteristics were well balanced including demographics and the previous medical history
- CAISR is the only one to assess the efficacy of neuroprotectants with intravenous Tenecteplase as the thrombolytic agent. Twenty seven patients were treated with Tenecteplase. All other trials have only recruited patients thrombolysed with Alteplase.
- Our mortality rates were lower at 10.2% and 14% in the Citicoline and placebo arms compared to 20% and 21% amongst the same respectively in the ICTUS trial
- The OR for a good functional outcome NIHSS or mRS of 0–2 was found to be 0.96 (95% CI 0.39–2.40) and 0.92 (95% CI 0.40–2.05). The ICTUS trial demonstrated an OR of 1.03 (95% CI 0.86–1.25) and 1.02 (95% CI 0.88–1.19) respectively.
- Fifteen patients in CAISR underwent endovascular treatment (7-Citicoline and 8-placebo). Such patients have previously never been studied for the effect of Citicoline. ESCAPE-NA1 trial [18] studied the effect of the neuroprotectant Nerinetide in this subgroup of patients. However, it was found to be ineffective in improving favourable clinical outcome.
- The median time of presenting to the hospital after the stroke was earlier in the Citicoline arm. Patients presented after 135 minutes, whereas they presented after 162.5 minutes in the control arm. This was 390 and 410 minutes respectively under the ICTUS trial, with 20% of patients receiving treatment after 12 hours
- The rates of hemorrhagic transformation were also similar with 6% of CAISR patients developing it compared to 8% in the ICTUS trial
- We tried to look for a surrogate marker of improvement in the form of stroke volume improvement. This was assessed by sequential MRIs done at baseline and 6 weeks after presentation to look for change in infarct size as a secondary outcome measure. The infarct volume decreased from week 1 to week 6 by 2.6 cm^3^ on placebo versus 4.2 cm^3^ on Citicoline. However, this was statistically insignificant. This is in comparison to S. Warach et al [15] who found a decrease in lesion volume from week 1 to week 12 by 6.9 cm^3^ on placebo versus 17.2 cm^3^ on Citicoline.
- Another neuroprotectant trial done on Nerinetide (ESCAPE-NA1) [18] during this time frame on patients with large vessel occlusions was found to be ineffective in promoting good functional outcome.

Failure to achieve primary outcome leads to many important questions [19]. One reason may be the ceiling effect of maximum benefit achieved by thrombolysis and mechanical thrombectomy. Another reason may be that the study was not powered enough (small sample size) to detect a meaningful difference in functional outcomes and also the study couldn’t achieve the planned sample size causing a reduction in power with a possibility of type 2 error. Even for the surrogate outcome of the stroke volume we could not achieve the sample size due to poor recruitment during COVID-19 pandemic. One reason for the same could be that lacunar stroke was the common stroke subtype in our patient set. We tried to see whether immediate peri-intervention administration of Citicoline and measurement of stroke volume at 6 weeks could show improvement in stroke volume, surrogate marker. It would have been justified to do a larger trial if we could at least show a definite or trend towards improvement in stroke volume.

## Conclusion

CAISR was the first to evaluate the role of Citicoline, when used immediately after recanalization therapy, when the penumbral tissue is the most susceptible either to be protected from injury or become ischemic. It was also the first to include posterior circulation strokes and patients undergoing mechanical thrombectomy. It is also the only trial on neuroprotectants to have recruited patients thrombolysed with Tenecteplase.

However, we did not find any significant difference between the Citicoline or placebo arms with respect to either our primary outcome (change in stroke volume) or secondary outcomes (NIHSS 0–2, mRS 0–2, Barthel index> = 95). We could not demonstrate benefit of Citicoline in our study population where it was used immediately after recanalization therapy.

## Supporting information

S1 File Abbreviations used. (DOCX)

S2 File Proforma of project proposal. (DOCX)

S1 Checklist Consort checklist. (DOC)
