# Supplementary material for: Citicoline in acute ischemic stroke: A randomized controlled trial
Source: PLoS One. 2022 May 31;17(5):e0269224. doi: 10.1371/journal.pone.0269224 (PMC9154187; doi:10.1371/journal.pone.0269224)
Supplement: S1 File — (DOCX) [file pone.0269224.s001.docx]

**Abbreviations used:**

ACA- Anterior Cerebral Artery

AIIMS- All India Institute of Medical Sciences

ASPECTS- Alberta Stroke Program Early CT Score

BD- Bis in die

BP- Blood Pressure

CI- Confidence Interval

CT- Computed Tomography

DWI- Diffusion-Weighted Imaging

EVT- Endovascular Thrombectomy

FLAIR- Fluid Attenuated Inversion Recovery

GCS- Glasgow Coma Scale

ICA- Internal Carotid Artery

ITT- Intention to treat

MCA- Middle Cerebral Artery

mRS- modified Rankin Scale

NCCT- Non-Contrast Computed Tomography

NIHSS- National Institute of Health Stroke Scale

NNT- Number needed to treat

OR- Odds Ratio

PCA- Posterior Cerebral Artery

rtPA- recombinant tissue plasminogen activator

SD- Standard Deviation

TNK- Tenecteplase

TOAST- Trial of Org10172 Acute Stroke Treatment
