# Supplementary material for: Citicoline in acute ischemic stroke: A randomized controlled trial
Source: PLoS One. 2022 May 31;17(5):e0269224. doi: 10.1371/journal.pone.0269224 (PMC9154187; doi:10.1371/journal.pone.0269224)
Supplement: S2 File — (DOCX) [file pone.0269224.s002.docx]

# CAISR CLINICAL TRIAL PROTOCOL

# PART I: GENERAL INFORMATION

**Category: Please put ‘X’ against the category under which the project should be considered.**

# Basic Research Clinical Research X

1. **Project Title**: Citicoline in Acute Ischemic stroke undergoing revascularization therapy (CAISR)
2. a. Broad Area: Clinical

b. b. Clinical Trial: Yes/No - **Investigator Initiated Academic Trial**

1. Duration: 2 years
2. Total Cost: 300,000 INR
3. Departments involved in the proposal: Neurology, Neuroradiology
4. Principal Investigator Name: Prof. M.V.Padma Srivastava

Date of Birth: 7.3.1965

Designation: Professor Department: Neurology

Telephone: +91-9810819167

E-mail: vasanthapadma123@gmail.com

1. a. Co-Investigator -I

Name: Ayush Agarwal

Date of Birth: 06.05.1988

Designation: Senior Resident Department: Neurology

Telephone: +91-8193900444

E-mail: [ayushthetaurian@gmail.com](mailto:ayushthetaurian@gmail.com)

7. b. Co-Investigator -II Name: Venugopalan Y. Vishnu

Date of Birth: 16.02.1984

Designation: Assistant Professor Department: Neurology

Telephone: 91-9855480361

E-mail: [vishnuvy16@yahoo.com](mailto:vishnuvy16@yahoo.com)

1. c. Co-Investigator -III

Name: Mamta Bhushan Singh Date of Birth: 6/9/1966 Designation: Professor Department: Neurology

Telephone:9818835305

E-mail: [mbsneuro@gmail.com](mailto:mbsneuro@gmail.com)

- 1. Co-Investigator -IV Name: Rohit Bhatia Date of Birth: 24.2.1971 Designation: Professor Department: Neurology

Telephone:9891267417

E-mail: [rohitbhatia71@yahoo.com](mailto:rohitbhatia71@yahoo.com)

- 1. Co-Investigator -V

Name: Vinay Goyal

Date of Birth: 20/05/1967

Designation: Professor Department: Neurology

Telephone:9868398216

E-mail: [drvinaygoyal@gmail.com](mailto:drvinaygoyal@gmail.com)

- 1. Co-Investigator –VI

Name: Ajay Garg

Date of Birth: 2/4/1971 Designation: Professor Department: Neuroradiology

Telephone:9868398217

E-mail: [drajaygarg@gmail.com](mailto:drajaygarg@gmail.com)

1. Project Summary (maximum 500 words):

Citicoline in acute ischemic stroke undergoing revascularization therapy is a randomized controlled trial aiming to detect whether citicoline during post intervention period of revascularization therapy in acute ischemic stroke patients will help in achieving a reduction in stroke volume at 3 months. Citicoline is a widely used neuroprotectant and is approved by DGCI for use in India. The current standard of care in acute ischemic stroke within window period is thrombolysis with or without endovascular therapy with stent retrievers. The role of neuroprotective therapies to salvage the ischemic penumbra is not clear. Most of the neuroprotective drug trials have included patients after the critical window period. Those patients undergoing revascularization therapy are expected to have a good ischemic penumbra. Our study is targeting this specific group of patients with intervention of citicoline immediately after the revascularization therapy so that the effect is maximum. Citicoline has never been used concurrently in any clinical trial involving endovascular therapy. The largest clinical trial on citicoline, ICTUS trial also did not have a single patient who underwent endovascular therapy. Moreover, no study has effectively studied the role of Citicoline in acute stroke immediately after or during revascularization (either thrombolysis or endovascular therapy).

The CAISR trial is a single center, prospective, randomized, open-label controlled trial with blinded outcome evaluation. It will be conducted at department of neurology and neuroradiology, All India Institute of Medical Sciences, New Delhi, India. We plan to include all acute stroke patients eligible for revascularization therapy (thrombolysis or endovascular thrombectomy) in the study. Informed consent will be taken for citicoline administration. The patients will be randomized after giving the standard of care treatment for acute stroke. Randomization will be performed using a computer-generated simple randomization sequence. In the citicoline arm, intravenous citicoline 1000mg will be given immediately after the revascularization therapy (thrombolysis/endovascular thrombectomy) and then every 12 hours for 3 days followed by oral citicoline 1000 mg every 12 hours for 6 weeks. The intravenous citicoline will be infused in a 100 ml saline solution bag and infused during 30–60 min. The control arm will receive 100 ml intravenous normal saline which will also be given immediately after the revascularization procedure and then every 12 hours for 3 days followed by oral multivitamin available in AIIMS pharmacy every 12 hours for 6 weeks. The primary outcome of the study is the reduction in stroke volume documented by MRI brain done within one hour of revascularization therapy and at 6 weeks. The secondary outcomes are MRS (0-2), NIHSS (0-2), Barthel Index >=95 at 90 days. Blinded outcome assessment will be done. The study will be conducted according to GCP guidelines and will be registered at clinical trials registry India. All adverse events and SAEs will be reported as per the GCP guidelines.


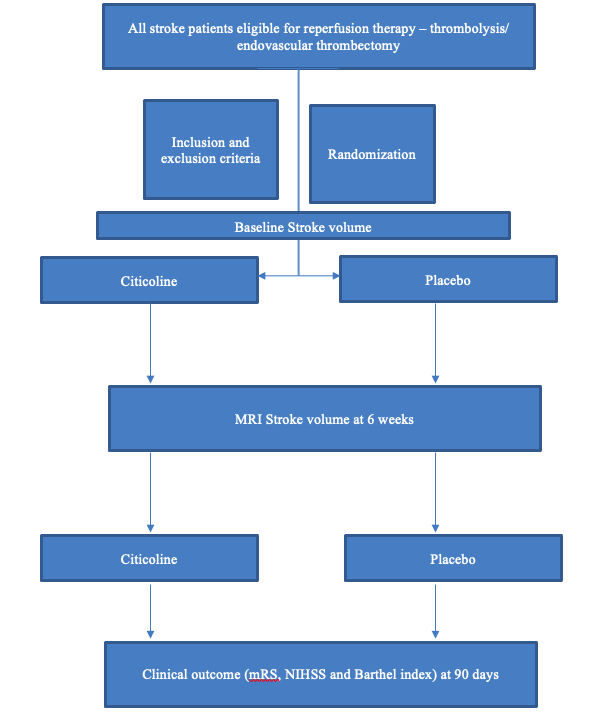


# PART II: TECHNICAL DETAILS OF PROJECT

1. **Research hypothesis**

Administration of citicoline immediately after reperfusion therapy (intravenous thrombolysis or endovascular thrombectomy) will salvage the ischemic penumbra in acute ischemic stroke patients.

# Research question

Whether administration of intravenous followed by oral citicoline in acute stroke patients immediately after reperfusion therapy (thrombolysis or endovascular thrombectomy), will help in achieving a reduction in stroke volume (MRI brain imaging) at 6 weeks and better clinical outcome at 12 weeks?

# Background and Rationale

The current standard of care in acute stroke within window period is intravenous thrombolysis and/or endovascular therapy with stent retrievers. ^1^ In the immediate management of acute stroke, apart from restoring brain perfusion by thrombolysis or endovascular thrombectomy, preservation of ischemic penumbra from further damage is a major research target.^2^The role of neuroprotectants in this scenario had been explored in various animal models and results were promising. But none of these drugs have been effective in phase 3 trials. One of the promising neuroprotectants is citicoline, an exogenous form of cytidine-5ʹ-diphosphocholine (CDP-choline) and an intermediate in phosphatidylcholine pathway.^3-5^ During ischemia, phosphatidylcholine is broken down into free fatty acids, which leads to production of free radicals that causes ischemic injury. In animal studies citicoline has been shown to decrease this cell membrane breakdown, causing increased phosphatidylcholine synthesis and decreased levels of free fatty acids. Moreover, citicoline treatment has been shown reduce infarct volume in animal models.

Citicoline is a widely used neuroprotectant in management of stroke. It is approved by DGCI for use in stroke and traumatic brain injury. Citicoline has been evaluated in various clinical studies.^6-8^ None of these studies showed any treatment efficacy. A meta-analysis of four placebo-controlled, double-blinded RCTs of citicoline in moderate-to-severe ischaemic stroke showed that citicoline improved recovery at 3 month follow-up^3^. This individual patient data pooled meta-analysis showed that moderate to severe stroke patients might benefit if citicoline was started within 24 hours of symptom onset.^4^Later a study- level meta-analysis showed some benefit of citicoline in disability reduction.^9^ The ICTUS trial was designed to confirm the findings of these meta-analysis.^5^ It had 2298 moderate to severe ischemic strokes patients who presented within 24 hours from symptom onset. But it was stopped in 2011 due to futility.

The primary outcome in ICTUS trial was recovery at 90 days assessed by a global test combining the favorable responses from three outcome scales: Barthel index (95–100), mRs (0–1), and NIHSS (0– 1). There was no difference in the 90-day global outcome end point (OR, 1.03; 95% CI, 0.86–1.25; P=0.364).^5^

Very few studies on citicoline have been conducted in India. Ghosh S et al studied the effect of citicoline on 100 patients of stroke (ischemic and hemorrhagic stroke).^10^ They showed that in stroke patients who present within 48 h of onset, management with citicoline improved the outcome (MRS, NIHSS and Barthel Index) at the end of both 1st and 3rd months. This was also observed in all stroke groups. None of the patients in this study received either thrombolysis or endovascular therapy. But this study was methodically weak. They did not provide details of randomization or concealment. Moreover, neither the treating physicians nor the outcome assessors were blinded to the drug allocation. Mitta M et al assessed the efficacy of edavarone and citicoline in 78 acute ischemic stroke (3-24 hours) patients.^11^ They included both anterior and posterior circulation strokes. The study had three arms- edavarone, citicoline and control groups. Edavarone group had better neurological outcome (MRS) at 3 months. They had excluded patients who presented within 3 hrs. None of the patients received any thrombolysis or endovascular therapy. Moreover, there was no blinding in allocation or outcome assessment. Hence it is difficult to make any strong conclusions from these studies. No study has been conducted in India which assessed the role of citicoline in patients undergoing reperfusion therapy.

# Stroke Volume

Citicoline has been shown to reduce the stroke volume in animal studies. In a double-blind placebo- controlled study on acute stroke patients (< 24 hours) conducted by Warach et al using diffusion weighted imaging (DWI), oral citicoline 500 mg/day was used for 6 weeks.^12^ They assessed the progression of stroke volume from baseline to 12 weeks. There was no change in the primary outcome (change in stroke volume) at 12 weeks in 81 patients studied. Even though a big difference in the percentage change of lesion size was found in favor of citicoline, the variance in the placebo group was also large, thereby preventing a statistically significant effect. There was a significant difference in reduction in stroke volume favoring citicoline in the secondary analysis. The authors attributed the negative results to small sample size and they assume that a sample size required to demonstrate an effect with a significance level of 0.05 and a power of 80% would be 58 per arm.

CASIR would be different from this study in the following aspects:

1. Includes all acute ischemic stroke patients undergoing reperfusion therapy, irrespective of the time frame
2. Uses intravenous citicoline followed by oral citicoline (as in ICTUS trial)
3. Citicoline is given immediately after the reperfusion therapy
4. The sample size is larger
5. Includes patients with both anterior and posterior circulation stroke
6. Patients with any deficit will be included

# Rationale

The role of citicoline in patients undergoing reperfusion strategies was never studied exclusively. CAISR is single center, prospective, randomized, open-label, controlled trial with blinded outcome evaluation, aiming to find the role of citicoline in acute stroke patients undergoing reperfusion therapy. The patients undergoing reperfusion therapy are a specific subgroup of stroke patients who may have a large ischemic penumbra and neuroprotectants may have an important role in salvaging those tissues. CAISR is targeting this specific group of patients with intervention of citicoline given immediately after reperfusion therapy so that the effect is maximum.

# Safety profile of Citicoline

The ICTUS trial showed that Citicoline is a safe drug with no significant adverse events.^4^ There was no significant difference in serious adverse events or other adverse events between citicoline and placebo arm. Mortality was comparable between the two groups (221 [19%] of 1148 patients in the citicoline group vs 242 (21%) of 1150 in the placebo group; p=0·31). Citicoline is approved by DGCI for use in stroke and traumatic brain injury.

1. **Preliminary work done if any:** Nil
2. **Objective:**

To find out whether administration of citicoline immediately after reperfusion therapy will cause reduction in stroke volume compared to standard treatment

1. **Detailed methodology including study design and outcome measures**

Study type: Interventional

Study design: Allocation: Randomized

Intervention Model: Parallel Assignment Masking: Blinded outcome assessment

Number of groups: 2 groups (Citicoline arm and Control arm)

Citicoline arm: Intravenous citicoline 1000mg during the revascularization therapy (thrombolysis/endovascular thrombectomy) and then every 12 hrs for 3 days followed by oral citicoline 1000 mg every 12 hrs for 6 weeks. The intravenous citicoline is infused in a 100 ml saline solution bag and infused during 60 min.

Control arm: Intravenous 100 ml normal saline will be given immediately after the revacularisation therapy (thrombolysis/endovascular thrombectomy) and then every 12 hrs for 3 days followed by oral multivitamin available in AIIMS pharmacy every 12 hrs for 6 weeks. The intravenous 100 ml saline solution will be infused in 60 min.


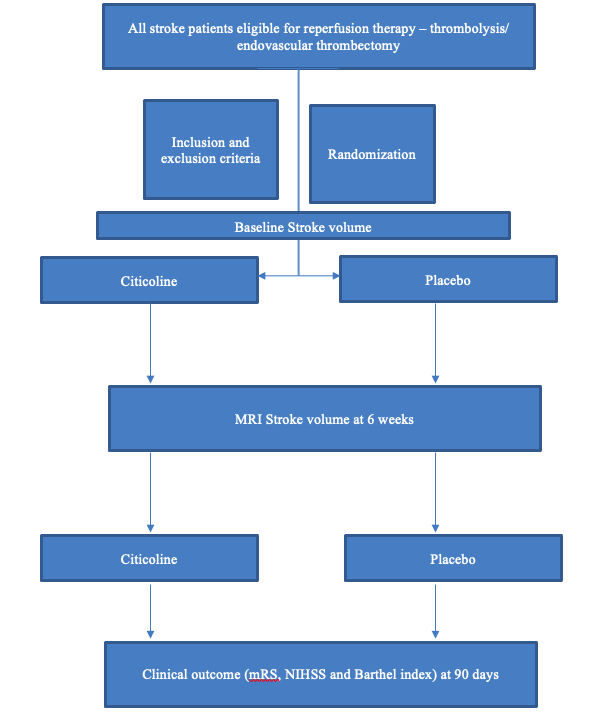


**Inclusion Criteria:**

1. Age >= 18 yrs
2. Suspected acute ischemic stroke based on clinical and radiographic evidence as determined and documented by the AIIMS stroke team
3. Participants must meet criteria for intravenous thrombolysis or intra-arterial thrombectomy as determined and documented by Stroke Neurologist and Interventional Neuroradiologist at AIIMS
4. Signed informed consent **Exclusion Criteria:**
5. Intracranial hemorrhage
6. Known allergic reactions to components Citicoline or contrast allergy
7. CT or conventional MRI evidence of brain tumor, subarachnoid and/or intracerebral and/or intraventricular hemorrhage
8. Previous disorders that may confound the interpretation of the neurological scales
9. Pre-existing dementia, when dementia implies a disability, measured as a score of 2 or higher in the previous MRS
10. Patients under current treatment with citicoline

# Outcome

# *Primary Outcome*

The primary outcome will be change in stroke volume from baseline to week 6.

# *Secondary Outcome*

MRS 0-2 at 90 days

NIHSS 0-2 at Day 90

Barthel Index >=95 at D90

# Imaging Protocol for Stroke Volume assessment

MRI brain will be done in any of the following machines: 3T Philips Achieva, 3T Ingenia, 1.5T GE optima. All images will be acquired in axial sequence. The MRI parameters assessed are as follows:

|  | 1.5T GE Optima | 3T Achieva | 3T Ingenia |
| --- | --- | --- | --- |
| DWI | TR: 6000 NOA: 2  TE:77 SBS: 0.5  ST: 5 Matrix: 128*128 | TR: 4050 NOA: 1  TE:96 SBS: 1  ST: 4 Matrix: 163*129 | TR: 3684 NOA: 1  TE:87 SBS: 1  ST: 4 Matrix: 152*122 |
| 3D T1W | TR: 8.47 NOA: 1  TE:3.27 SBS: 0  ST: 1 Matrix: 256*256 | TR: 8.2 NOA: 1  TE:3.74 SBS: 0  ST: 1 Matrix: 240*240 | TR: 8 NOA: 1  TE:3.6 SBS: 0  ST: 1 Matrix: 240*240 |
| 3D FLAIR | TR: 6500 NOA: 1  TE:92 SBS: 0  ST: 1.2 Matrix: 256*224 | TR: 4800 NOA: 2  TE:275 SBS: 0  ST: 1 Matrix: 240*240 | TR: 4800 NOA: 2  TE:300 SBS: 0  ST: 1 Matrix: 240*240 |
| SWI | TR: 74 NOA: 0.7  TE:47.5 SBS: 0.8 ST: 1.6 Matrix: 320*256 | TR: 31 NOA: 1  TE:0 SBS: 0  ST: 0.9 Matrix: 384*384 | TR: 6000 NOA: 2  TE:77 SBS: 0.5  ST: 5 Matrix: 128*128 |
| TOF MRA | TR: 19 NOA: 1  TE:2.8 SBS: 0.6  ST: 1.2 Matrix: 320*192 | TR: 14.84 NOA: 1  TE:3.45 SBS: 0.75 ST: 1.5 Matrix: 380*243 | TR: 23 NOA: 1  TE:3.45 SBS: 0.7 ST: 1.4 Matrix: 400*266 |

NOA: Number of averages SBS: Space between slices ST: Slice thickness

# Data analysis plan

The primary analysis will be an intention to treat analysis. The assumption is that the lesion volume would decrease in 39% of the placebo arm and 65% of the citicoline-treated patients, we estimated that 116 (58 per group) completed patients (baseline and week six MRI assessments completed) were required to detect this difference with 80% power.

# References

1 . Powers WJ, Derdeyn CP, Biller J et al; American Heart Association Stroke Council. 2015 American Heart Association/American Stroke Association Focused Update of the 2013 Guidelines for the Early Management of Patients With Acute Ischemic Stroke Regarding Endovascular Treatment: A Guideline for Healthcare Professionals From the American Heart Association/American Stroke Association. Stroke.

2015 Oct;46(10):3020-35.

2. del Zoppo GJ. Stroke and neurovascular protection. N Engl J Med 2006; 354: 553–55.

3 . Dávalos A, Secades J. Citicoline preclinical and clinical update 2009–2010. Stroke 2011; 42: S36–39.

1. Davalos A, Castillo J, Alvarez-Sabin J, et al. Oral citicoline in acute ischemic stroke: an individual patient data pooled analysis of clinical trials. *Stroke* 2002; **33:** 2850–57.
2. Dávalos A, Alvarez-Sabín J, Castillo J, Díez-Tejedor E, Ferro J, Martínez-Vila E, Serena J, Segura T, Cruz VT, Masjuan J, Cobo E, Secades JJ; International Citicoline Trial on acUte Stroke (ICTUS) Trial Investigators. Citicoline in the treatment of acute ischaemic stroke: an international, randomised, multicentre, placebo-controlled study (ICTUS trial). Lancet. 2012;380:349–357.
3. Clark WM, Warach SJ, Pettigrew LC, Gammans RE, Sabounjian LA. A randomized dose-response trial of citicoline in acute ischemic stroke patients: Citicoline Stroke Study Group. Neurology. 1997;49:671–678.
4. Clark WM, Wechsler LR, Sabounjian LA, Schwiderski UE; Citicoline Stroke Study Group. A phase III randomized efficacy trial of 2000 mg citicoline in acute ischemic stroke patients. Neurology. 2001;57:1595–1602.
5. Clark WM, Williams BJ, Selzer KA, Zweifler RM, Sabounjian LA, Gammans RE. A randomized efficacy trial of citicoline in patients with acute ischemic stroke. Stroke. 1999;30:2592–2597.
6. Saver JL. Citicoline: update on a promising and widely available agent for neuroprotection and neurorepair. Rev Neurol Dis. 2008;5:167–177.
7. Ghosh S, Das SK, Nath T, Ghosh KC, Bhattacharyya R, Mondal GP. The effect of citicoline on stroke: A comparative study from the Eastern part of India. Neurol India. 2015 Sep-Oct;63(5):697-701.
8. Mitta M, Goel D, Bansal KK, Puri P. Edaravone - citicoline comparative study in acute ischemic stroke (ECCS-AIS). J Assoc Physicians India. 2012 Nov;60:36-8.
9. Warach S, Pettigrew LC, Dashe JF et al. Effect of Citicoline on Ischemic Lesions as Measured by Diffusion-Weighted Magnetic Resonance Imaging. Ann Neurol 2000;48:713–722
